# Relationships between the Reversible Oxidation of the Single Cysteine Residue and the Physiological Function of the Mitochondrial Glutaredoxin S15 from *Arabidopsis thaliana*

**DOI:** 10.3390/antiox12010102

**Published:** 2022-12-31

**Authors:** Loïck Christ, Jérémy Couturier, Nicolas Rouhier

**Affiliations:** 1 Université de Lorraine, INRAE, IAM, F-54000 Nancy, France; 2 Institut Universitaire de France, F-75000 Paris, France

**Keywords:** glutathione, glutaredoxin, cysteine oxidation, mitochondria

## Abstract

Glutaredoxins (GRXs) are widespread proteins catalyzing deglutathionylation or glutathionylation reactions or serving for iron-sulfur (Fe-S) protein maturation. Previous studies highlighted a role of the *Arabidopsis thaliana* mitochondrial class II GRXS15 in Fe-S cluster assembly, whereas only a weak glutathione-dependent oxidation activity was detected with the non-physiological roGFP2 substrate in vitro. Still, the protein must exist in a reduced form for both redox and Fe-S cluster binding functions. Therefore, this study aimed at examining the redox properties of *At*GRXS15. The acidic pKa of the sole cysteine present in *At*GRXS15 indicates that it should be almost totally under a thiolate form at mitochondrial pH and thus possibly subject to oxidation. Oxidizing treatments revealed that this cysteine reacts with H_2_O_2_ or with oxidized glutathione forms. This leads to the formation of disulfide-bridge dimers and glutathionylated monomers which have redox midpoint potentials of −304 mV and −280 mV, respectively. Both oxidized forms are reduced by glutathione and mitochondrial thioredoxins. In conclusion, it appears that *At*GRXS15 is prone to oxidation, forming reversible oxidation forms that may be seen either as a catalytic intermediate of the oxidoreductase activity and/or as a protective mechanism preventing irreversible oxidation and allowing Fe-S cluster binding upon reduction.

## 1. Introduction

Glutaredoxins (GRXs) are ubiquitous proteins belonging to the thioredoxin (TRX) superfamily [1]. They adopt a canonical tridimensional structure composed of a central β-sheet formed by four β-strands classically surrounded by three α-helices, but one or two extra α-helices are sometimes present [2]. A cysteine or a pair of cysteines present in a CxxC/S motif is normally present in the loop preceding the helix α1. Most organisms possess two GRX classes, referred to as classes I and II, depending on their active site motifs and primary sequences [3].

Class I GRXs contain a CxxC/S active site motif and generally act as oxidoreductases [4,5], catalyzing protein glutathionylation/deglutathionylation reactions [6,7,8]. Noteworthy, some of them are able to bind an iron-sulfur (Fe-S) cluster, which is thought to serve for sensing oxidative conditions [9,10,11,12]. For their activity, redox active class I GRXs usually require a single catalytic cysteine even though one or two additional cysteines are sometimes present [5,10,13]. A recent study proposed the existence of two different glutathione recognition sites. A glutathione scaffold site allows the recognition of glutathionylated substrates [14]. This drives the oxidative half-reaction which consists in the nucleophilic attack of the catalytic cysteine thiolate on the glutathionylated substrate and results in the formation of a disulfide bridge between the GRX and the glutathione molecule. Then, the reductive half-reaction is achieved by a reduced glutathione (GSH) molecule that would bind in a so-called GSH activator site; however, evidence for the involved residues are currently lacking [14]. After this step, GRXs are reduced, and an oxidized glutathione (GSSG) is formed but is efficiently reduced back to GSH by NADPH-dependent glutathione reductases. This catalytic mechanism is referred to as a monothiol mechanism since it involves only one cysteine of the GRX moiety. When the catalytic mechanism involves a second cysteine of the GRXs, for instance when GRXs catalyze the reduction of disulfide bonds formed between two cysteine residues of the partner proteins, it is referred to as a dithiol mechanism [5,15]. The second cysteine may be present either in the active site CxxC signature or in the C-terminal part of the protein. It is referred to later on as a semi-conserved cysteine since it is present in many class I or II GRXs but not all. In both cases, this leads to the formation of an intramolecular disulfide bridge that is reduced by GSH, TRXs, or TRX reductases depending on the cases [15,16,17,18].

Class II GRXs contain a very conserved CGFS signature and eventually the semi-conserved cysteine in the C-terminal region. They usually have no or little oxidoreductase activity [15,16,17,19,20] and they may be uniquely involved in the maturation of Fe-S proteins via their capacity to bind and transfer a [2Fe-2S] cluster [12]. In both class I and class II Fe-S cluster binding GRXs, the Fe-S cluster is usually bridged into homodimers via the first cysteine of the CxxC motif and the cysteine of two GSH molecules [12]. The different properties between class I and class II GRXs, i.e., redox activity vs. Fe-S cluster binding and transfer properties, have been the subject of intense research [14,21,22]. Some differences at the level of the loop preceding the active site motif (elongated in class II GRXs), of the active site motif itself (Gly and Phe in CGFS active site motif of class II GRXs vs. Pro/Ser and Tyr at the equivalent position in class I GRXs), or of the helix α3 and following loop (basic residues in class I GRXs replaced by acidic or hydrophobic residues in class II GRXs) are thought to preclude recognition of glutathionylated substrates and/or deglutathionylation activity of class II GRXs and therefore to prevent their oxidoreductase activity.

The *Arabidopsis thaliana* GRXS15 (*At*GRXS15) belongs to the class II GRXs and is the sole GRX present in mitochondria [19]. It is a protein essential for the maturation of Fe-S proteins in this organelle by receiving the preformed Fe-S cluster built up on the so-called ISCU scaffold proteins and transferring it notably to ISCA maturation factors or to acceptor proteins such as the mitochondrial ferredoxin 1 [19,23,24,25]. Using the roGFP2 as a tool for measuring the oxidoreductase activity in vitro, it was previously reported that *At*GRXS15 does not have the capacity to reduce roGFP2 in the presence of GSH, whereas a weak oxidation activity was observed in the presence of GSSG [14,19]. This suggested that the reductive half-reaction is altered in *At*GRXS15 but also raised the question of why roGFP2 is reduced when expressed in plant mitochondria [26,27]. Unlike most other class II GRXs and in particular the yeast and human GRX5 orthologs, *At*GRXS15 only possesses the cysteine present in the CGFS signature, which suggests that the reaction mechanism and/or oxidation forms adopted by *At*GRXS15 might be different from other class II GRXs. In yeast Grx5, the presence of the semi-conserved C-terminal cysteine allows the formation of an intramolecular disulfide in the apoform that is reduced by GSH albeit with a low efficiency [20], but also the binding of a [4Fe-4S] cluster into a dimer without relying on glutathione ligands [28]. Regardless of their capacity to catalyze oxidoreduction reactions or to bind an Fe-S cluster, an important prerequisite for both functions is that GRXs exist initially in a reduced form. However, they are present in subcellular compartments where oxidative reactions occur. In fact, in a study investigating the changes in the redox metabolism upon seed imbibition, *At*GRXS15 was identified as one of the mitochondrial proteins that undergoes significant reduction, indicating first that it is prone to oxidation and second that there is likely a NADPH-dependent pathway operating for reduction [29]. For the reasons detailed above, we sought to analyze the redox properties of a recombinant *At*GRXS15. In particular, we measured the pKa of its single cysteine residue and its sensibility to various oxidants. Two oxidation forms, a glutathionylated monomer and an intermolecular disulfide-bridged dimer with close midpoint redox potentials, have been detected and shown to be reduced by GSH or mitochondrial TRXs o.

## 2. Materials and Methods

### 2.1. Heterologous Expression in E. coli and Protein Purification

The cloning of the sequence coding for the mature form of *A. thaliana* GRXS15 (AT3G15660) into the bacterial pET3d expression vector was previously described [24]. The *E. coli* C41(DE3) strain containing the helper pSBET plasmid [30] was used for protein expression. Bacteria were grown at 37 °C in a total volume of 3.2 L of LB medium, supplemented with 50 µg/mL of kanamycin and ampicillin. Protein expression was induced at the exponential phase by adding 100 µM of isopropyl β-D-1-thiogalactopyranoside and bacterial cultures were further incubated at 37 °C for 5 h. Cells were harvested by centrifugation (Beckman Coulter JLA 8.1000; 20 min; 6200× *g*; 20 °C) and pellets were resuspended in 12 mL buffer A containing 30 mM Tris-HCl pH 8.0, 200 mM NaCl, and 1 mM EDTA and stored at −20 °C. Cell lysis was performed by sonication (20% amplitude; 3 times 1 min), then soluble and insoluble fractions were separated by centrifugation (Beckman Coulter JA 25.50; 20 min; 39,000× *g*; 4 °C). The soluble fraction was precipitated by adding ammonium sulfate up to 30% and then 80% of the saturation in two successive steps separated by centrifugation (JA 25.50; 20 min; 39,000× *g*; 4 °C). Proteins having precipitated between 30 and 80% of the ammonium sulfate saturation were resuspended in 8 mL buffer B (30 mM Tris-HCl pH 8.0, 200 mM NaCl) and incubated for 1 h with 20 mM EDTA and 2.5 mM potassium ferricyanide at 4 °C. The protein sample was then loaded on size exclusion chromatography (SEC) (ACA 44; Pall-BioSepra) equilibrated with buffer A. Fractions containing the protein of interest were pooled and dialyzed against buffer C containing 30 mM Tris-HCl pH 8.0 and 1 mM EDTA and incubated for 1 h with 10 mM DTT at 4 °C before loading on Q-sepharose column equilibrated with buffer C. Retained proteins were then eluted using a linear 0–400 mM NaCl gradient. Interesting fractions were pooled, concentrated in 1 mL using an Amicon cell equipped with YM10 membrane, and loaded on a Superdex S75 16/600 column equilibrated with buffer B and coupled to a FPLC system (Äkta purifier). Fractions containing monomeric *At*GRXS15 were then concentrated. Sample purity was assessed through SDS-PAGE and sample concentration was determined spectrophotometrically using a theoretical molar extinction coefficient at 280 nm of 8480 M^−1^.cm^−1^. The other proteins used in this work, *A. thaliana* TRXo1, TRXo2, and NADPH-dependent thioredoxin reductase B (NTRB) were purified as previously described [31,32].

### 2.2. pKa Determination

For its reduction, *At*GRXS15 (200 µM) was incubated with a 20-fold molar excess of TCEP for 1 h at 4 °C before desalting using Zeba spin columns (Thermo Fischer Scientific, Waltham, MA, USA). Pre-reduced *At*GRXS15 (10 µM) was incubated in buffers ranging from pH 2.0 to 8.0 (100 mM citrate buffers are used from pH 2.0 to 6.0, and 100 mM phosphate buffers from pH 6.5 to 8.0) containing 200 µM 5-iodoacetamido-fluorescein (5-IAF) for 15 min at room temperature (RT) in the dark [33]. Proteins were then precipitated with 10% tricholoroacetic acid (TCA) and, after 15 min at 4 °C, centrifuged at 13,000× *g* for 10 min at 4 °C. The pellets were washed with pure acetone and centrifuged at 13,000× *g* for 10 min at 4 °C. After resuspension in buffer D containing 100 mM Tris-HCl pH 8.0 and 1% SDS, fluorescence emission at 518 nm of each sample was measured for an excitation wavelength of 494 nm using a Variant Cary Eclipse fluorimeter. Gain settings to detect 5-IAF-labeled thiolate groups were adjusted before each experiment, therefore absolute values were transformed into %_fluorescence_ with respect to the maximum and minimum values within the series using the following equation:

%_fluorescence_ = (fluorescence_value_ − fluorescence_min_)/(fluorescence_max_ − fluorescence_min_) × 100.

### 2.3. Sensibility to Oxidants

For oxidation reactions, pre-reduced *At*GRXS15 (13 µM) was incubated with 5- and 10-fold excess of the oxidizing agent (H_2_O_2_, GSSG, GSNO) for 2 h at RT. Proteins were then precipitated with 10% TCA and treated as described above (Section 2.2). After acetone evaporation, pellets were resuspended in buffer D supplemented with 2 mM mPEG24 and incubated for 10 min at RT. Then, samples were loaded on non-reducing SDS-PAGE. For quantitative assays, pre-reduced protein (5 µM) was incubated with up to 10 mM of oxidants (H_2_O_2_, GSSG, GSNO) in buffer B for 1 h at RT. After this incubation period, mBBr (100 µM) was added to all samples and further incubated for 20 min in the dark. Proteins were then precipitated on ice with 10% TCA and treated as described above (Section 2.2). Once acetone evaporated, the pellets were resuspended in buffer D and then fluorescence emission at 472 nm of each sample was measured for an excitation wavelength of 380 nm using a Variant Cary Eclipse fluorimeter. Gain settings to detect mBBr-labelled thiol groups were adjusted before each experiment, therefore absolute values were transformed into %_fluorescence decrease_ to the respective initial value of pre-reduced protein using the following equation:

%_fluorescence decrease_ = 100 − ((measured fluorescence− fluorescence_min_)/(fluorescence_max_ − fluorescence_min_) × 100). (1)

### 2.4. Redox Potential Determination of Oxidized Forms

To obtain dimeric or glutathionylated *At*GRXS15, reduced *At*GRXS15 (200 µM) was incubated for 2 h either with a 10-fold excess of H_2_O_2_ (2 mM) or with 10 mM GSH followed by the addition of 6 mM H_2_O_2_, respectively. After reaction, samples were desalted using the Zeba Spin desalting column. Then, pre-oxidized proteins (10 µM) were incubated for 2 h in 200 µL of a 100 mM HEPES pH 7.0 buffer containing various ratios of DTT/DTT_ox_ at a final concentration of 2 mM with defined redox potentials ranging from −220 mV to −400 mV [33]. After incubation, mBBr (2.5 mM) was added to each sample and further incubated for 20 min in the dark. Proteins were then precipitated on ice with 10% TCA and treated as described above (Section 2.2). Pellets were resuspended in buffer D, and fluorescence emission at 472 nm of each sample was measured for an excitation wavelength of 380 nm using a Variant Cary Eclipse fluorimeter. Gain settings to detect mBBr-labelled thiol groups were adjusted before each experiment, therefore absolute values were transformed into %_fluorescence_, taking into account that proteins were 100% reduced in buffer with the more electronegative E_m_ value and 100% oxidized in buffer with the less electronegative E_m_ value.

### 2.5. Reduction Tests

Dimeric and glutathionylated proteins obtained as described above (Section 2.4) were incubated with two different reducing systems. The thioredoxin system included 200 µM NADPH, 160 nM *A. thaliana* NTRB, and 2.5 µM of mitochondrial *At*TRXo1 or *At*TRXo2. Reduction by glutathione was assessed using GSH concentrations comprised between 25 µM and 50 mM. Reactions were performed in 50 µL of 30 mM Tris-HCl pH 8.0 and started by adding pre-oxidized *At*GRXS15 (13 µM). After 15 or 60 min of incubation at RT, the reaction was stopped by adding 10% TCA and treated as described above (Section 2.2). The acetone-washed protein pellets were resuspended in buffer D supplemented with 2 mM mPEG24 and further incubated for 10 min at RT. Then, each sample was separated on non-reducing SDS-PAGE. For the GSH treatments, band intensities were quantified using an Odyssey gel scanner (LiCor) and corrected by subtraction of the background. The values were transformed as percentages of oxidized forms reduced, with the minimal and maximal values corresponding to 0 and 100%. The normalized percentages were plotted against GSH concentration.

### 2.6. Replicates

All results represent mean ± standard deviation of at least three independent experiments. For SDS-PAGE, a gel representative of at least three independent experiments is shown.

## 3. Results and Discussion

### 3.1. The Cysteine of AtGRXS15 Has an Acidic pKa

**Figure 1:**
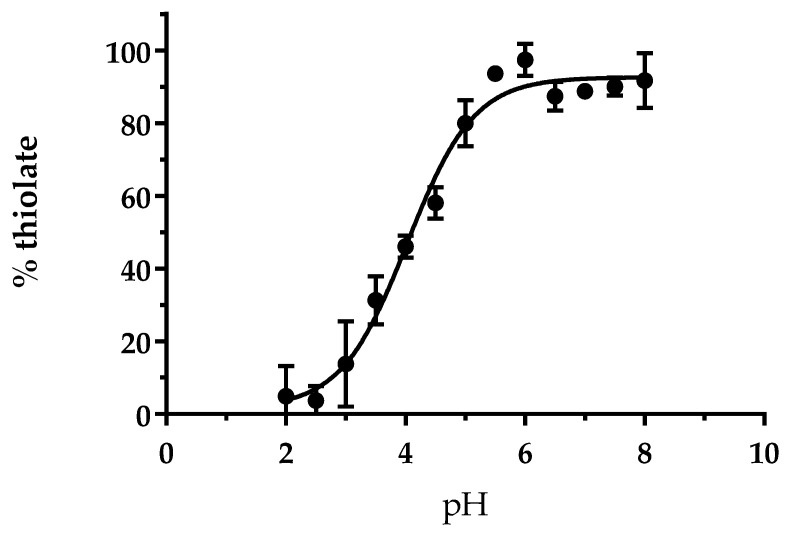
pKa determination of the single cysteine of *At*GRXS15. Pre-reduced recombinant *At*GRXS15 (10 µM) was incubated for 15 min with 200 µM 5-iodoacetamido-fluorescein (5-IAF) in buffers ranging from pH 2.0 to 8.0. Fluorescence emission values were then transformed into % thiolate and the values plotted against pH. Data are represented as mean ± SD of three independent experiments.

Previous studies using the classical hydroxyethyldisulfide (HED) and roGFP2 reduction assays demonstrated the absence of reductase activity for *At*GRXS15 using these two artificial substrates but a weak glutathionylation activity with roGFP2 [14,19]. Considering that the oxidoreductase activity notably relies on the reactivity of catalytic cysteine, we decided to determine the pKa of Cys91. Reduced *At*GRXS15 was incubated in different buffers ranging from pH 2.0 to 8.0 with 5-iodoacetamido-fluorescein, an alkylating agent able to react with thiolate but not thiol groups, thus allowing to discriminate both forms. From this experiment, we determined a pKa value of 4.0 ± 0.1 (Figure 1). This pKa value is similar to those determined for the active site cysteine of class I GRXs from poplar [5,34,35] or *Chlamydomonas reinhardtii* [36], which ranged from 3.9 to 5.3, but also of class II GRXs as shown for *A. thaliana* GRXS16 (pKa~3.9) [15], and the yeast ortholog Grx5 (pKa~5.0) [20]. Overall, this indicates that Cys91 of *At*GRXS15 should predominantly exist under a thiolate form at physiological pH and should be reactive. While this might not be sufficient to confer an oxidoreductase activity with large protein substrates because other biochemical or structural features matter, it may make the protein prone to oxidation by small oxidizing agents.

### 3.2. AtGRXS15 Is Prone to Oxidation by Physiological Oxidants

**Figure 2:**
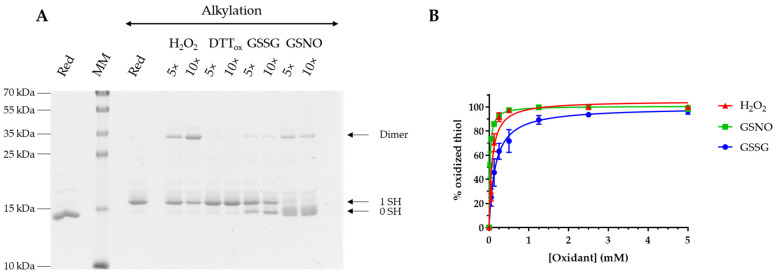
Sensibility of *At*GRXS15 towards oxidants. Pre-reduced recombinant *At*GRXS15 (13 µM) was incubated with a 5- to 10-fold molar excess of H_2_O_2_, DTT_ox_, GSSG, and GSNO for 2 h at RT. Remaining reduced thiols were then alkylated before protein migration on non-reducing SDS-PAGE. The numbers on the right correspond to the number of thiols that remained reduced upon treatment and thus were alkylated. A gel representative of at least three independent experiments is shown here (**A**). In another series of experiments, pre-reduced recombinant *At*GRXS15 (5 µM) was incubated for 1 h at RT with H_2_O_2_, GSSG, and GSNO at concentrations ranging from 50 µM to 10 mM. The remaining free thiols were alkylated with mBBr and quantified through measurement of the resulting fluorescence. The decrease of fluorescence, represented as a percentage of oxidized thiol, was plotted against the oxidant concentration for determination of S_0_._5_ value (**B**). Data are represented as mean ± SD of three independent experiments.

To assess the sensitivity of *At*GRXS15 to oxidation, we treated the reduced protein with a 5- and 10-fold excess of H_2_O_2_, oxidized DTT (DTT_ox_), GSSG, and GSNO for 2 h. Afterwards, the remaining free thiols were alkylated with mPEG24 to separate oxidized and reduced forms on non-reducing SDS-PAGE. As a reference, a shift of 1.2 kDa due to mPEG24 alkylation is visible when comparing the migration of a reduced monomer that is alkylated or not. Unlike DTT_ox_ which did not promote the oxidation of *At*GRXS15, an H_2_O_2_ treatment mainly induced the oxidation of *At*GRXS15 under a dimeric form whereas treatments with GSSG and GSNO mainly generated a monomeric oxidized form, although dimers are formed to some extent. It can be noted that a certain fraction of reduced protein remained upon H_2_O_2_ or GSSG treatments, which is not observed with GSNO (Figure 2A). Overall, this indicates that at these concentrations and excess, the oxidation of *At*GRXS15 monomers is not that efficient which allows dimerization. Whatever the oxidant used, dimer formation should thus be the result of a nucleophilic attack of a thiolate group from a reduced monomer on either a sulfenic acid, or a glutathionylated or nitrosylated cysteine. 

Further experiments were performed to obtain more quantitative data about *At*GRXS15 oxidation. Reduced *At*GRXS15 was incubated for 1 h in the presence of increasing concentrations of oxidants and then reacted with mBBr, a fluorescent alkylating reagent specific of thiol groups. The observed decrease of fluorescence, which reflects an increase of *At*GRXS15 oxidation, is represented as a percentage of thiol oxidation that was plotted as a function of oxidant concentration (Figure 2B). This allowed us to calculate S_0_._5_ values that correspond to the concentrations of oxidant necessary to reach half-maximal protein oxidation. These S_0_._5_ values are of 72 ± 10 µM for H_2_O_2_, 154 ± 14 µM for GSSG, and 21 ± 1 µM for GSNO, indicating that *At*GRXS15 is more sensitive to GSNO than to H_2_O_2_ and to GSSG. In comparison with similar studies performed with *At*GRXS16, *At*GRXS15 appears more susceptible to H_2_O_2_, approximately as sensitive to GSNO, and less susceptible to GSSG [15]. The major difference is that *At*GRXS16 possesses an additional semi-conserved cysteine in the C-terminal region, which leads to the formation of an oxidized form with an intramolecular disulfide bridge. This may also account for the better efficiency of *At*GRXS16 in catalyzing the GSSG-dependent oxidation of roGFP2 [14,15,19]. Similarly, the presence of an additional cysteine in the C-terminal part of other class II GRXs, *Ec*Grx4 [16], *Sc*Grx5 [20], and *Cr*GRX3 [17] for instance, also leads to the formation of an intramolecular disulfide bridge. All these results suggested that the presence of the C-terminal cysteine prevents the covalent dimerization. However, dimerization of *C. reinhardtii* GRX5 and GRX6, two class II GRXs, was observed upon DTT_ox_ treatment [36] and in the case of *Cr*GRX5, the C-terminal cysteine is present, indicating that there might be other determinants controlling dimerization.

### 3.3. The AtGRXS15 Intermolecular Disulfide Bonds Have Low Redox Potentials

**Figure 3:**
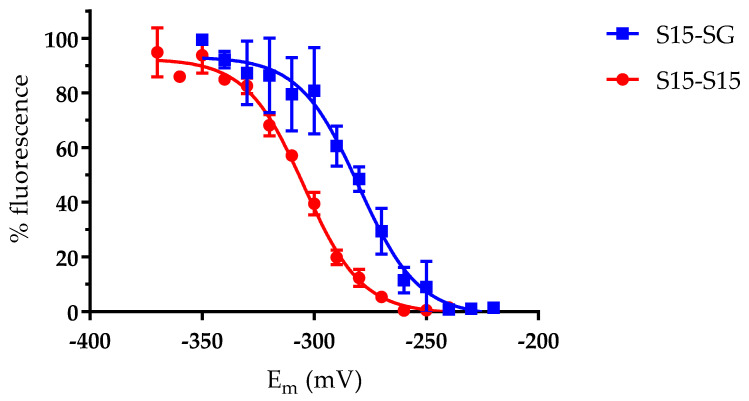
Redox midpoint potentials of glutathionylated and disulfide-bridged forms of *At*GRXS15. After incubation of pre-oxidized recombinant *At*GRXS15 (10 µM) forms (either glutathionylated monomer or oxidized dimer) in mixtures of DTT_red_/DTT_ox_ with defined redox potentials, reduced thiols were labelled with mBBr. Fluorescence emission corresponding to mBBr-labelled thiols was then transformed to a % fluorescence (relatively to the minimum and maximum values obtained), which was plotted against the respective redox potentials. Data are represented as mean ± SD of three independent experiments.

In vitro oxidation experiments suggested that two thiol/disulfide couples could be considered for *At*GRXS15, one for the intermolecular disulfide bridge formed upon *At*GRXS15 dimerization (2R-S^−^/R-SS-R) and one for a glutathionylated monomer (R-S^−^/R-SSG) because both GSSG and GSNO could oxidize *At*GRXS15. While we aimed at identifying potential differences in the redox properties of both oxidized forms, by determining their redox midpoint potentials (E_m_), glutathionylation by GSSG and GSNO was not complete. Hence, we applied a treatment combining H_2_O_2_ and GSH, which was previously reported to induce glutathionylation of *A. thaliana* glyceraldehyde-3-phosphate dehydrogenase (*At*GAPDH) for instance [37]. Incidentally, in a physiological context, the sulfenic acid would preferentially be attacked by GSH rather than by another reduced *At*GRXS15. Indeed, this treatment proved to work well since a single oxidized monomeric species that is reduced in the presence of reductants was obtained. Titration experiments using both types of pre-oxidized proteins incubated in various mixtures of DTT_red_/DTT_ox_ demonstrated low E_m_ values of −280 ± 2 mV and −304 ± 1 mV at pH 7.0 for the glutathionylated monomer and disulfide-bridged dimer, respectively (Figure 3).

Several oxidized GRX forms have been previously described either for class I or class II GRXs and E_m_ values reported in some cases. Concerning the redox potential of the glutathione adduct, it has been shown to oscillate from −242 mV to −254 mV at pH 7.0 for dithiol poplar class I GRXs [5] and even to reach −315 mV at pH 7.0 for poplar GRXS12, an atypical monothiol class I GRX [35]. The value obtained for the class II *At*GRXS15 (−280 mV) is situated in the middle. In fact, most class II GRXs possess the C-terminal semi-conserved cysteine which forms intramolecular disulfide bonds with the active site cysteine of the CGFS motif. The redox potentials for these intramolecular disulfides range from −175 mV at pH 7.0 for *Sc*Grx5 [20] to −323 mV at pH 7.9 (which translates into −269 mV at pH 7.0) for *Cr*GRX3 and to −296 mV at pH 7.0 for *At*GRXS16 [15,17]. To the best of our knowledge, the formation of an intermolecular disulfide involving the active site cysteine of class II GRXs has not been reported so far, which prevents comparison of the redox potentials. The only information is that an intermolecular disulfide bond formed on homodimeric poplar GRXC1 and GRXC2 and involving the semi-conserved C-terminal cysteine of two monomers can be reduced by GSH [5].

### 3.4. Oxidized Forms of GRXS15 Are Reduced by Mitochondrial Reduction Systems

**Figure 4:**
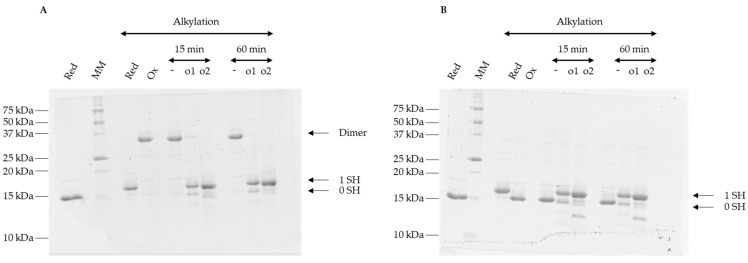
Reduction of oxidized *At*GRXS15 forms by mitochondrial thioredoxins. Pre-oxidized recombinant *At*GRXS15 (13 µM), either dimeric (**A**) or glutathionylated (**B**), was incubated with the reconstituted mitochondrial TRX system (*At*TRX o1 or o2, *At*NTRB, NADPH) for 15 or 60 min. Remaining reduced thiols were then alkylated before sample migration on non-reducing SDS-PAGE. The numbers on the right correspond to the number of thiols that remained reduced upon treatment and thus were alkylated. Gels are representative of at least three independent experiments.

The low redox midpoint potential values of the disulfide bonds formed on glutathionylated and dimeric *At*GRXS15 led us to evaluate the capacity of GSH or mitochondrial TRXs to reduce these species. As demonstrated by the appearance of a band at 17 kDa corresponding to the reduced *At*GRXS15 monomer, both mitochondrial thioredoxins o (*At*TRXo1 and *At*TRXo2) had the ability to reduce *At*GRXS15 homodimers and glutathionylated monomers (Figure 4A,B). 

**Figure 5:**
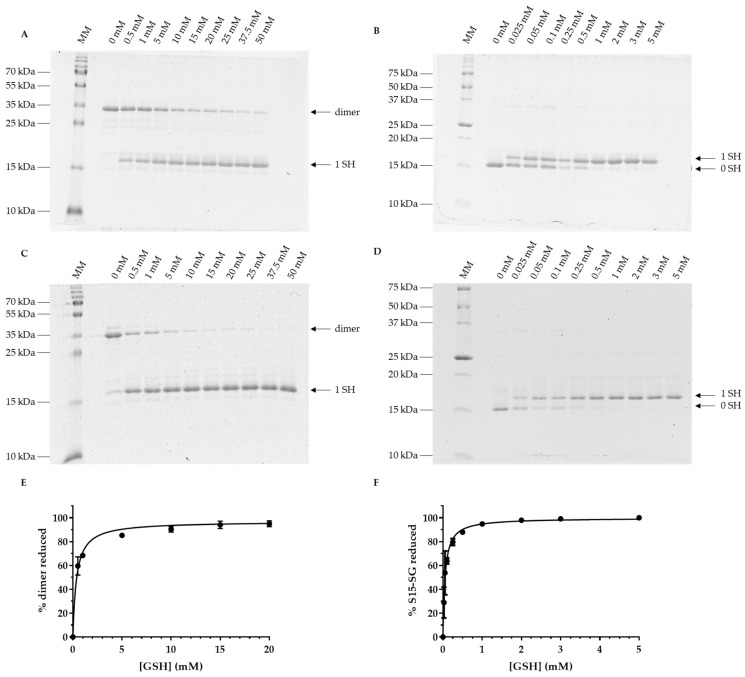
Reduction of oxidized *At*GRXS15 forms by GSH. Pre-oxidized recombinant *At*GRXS15 (13 µM), either dimeric (**A**,**C**) or glutathionylated (**B**,**D**), was incubated with 25 µM to 50 mM of GSH for 15 min (**A**,**B**) or 60 min at RT (**C**,**D**). Remaining reduced thiols were then alkylated before sample migration on non-reducing SDS-PAGE. The numbers on the right correspond to the number of thiols that were reduced upon treatment and thus were alkylated. Because the redox equilibrium between oxidized *At*GRXS15 and GSH was only complete for 60 min of incubation, gel bands were quantified for dimeric (**E**) and glutathionylated (**F**) *At*GRXS15 only at this time-point. The normalized intensities were transformed into % oxidized forms reduced respective to the minimum and maximum obtained and those values were plotted against GSH concentration. Gels are representative of at least three independent experiments and thus quantification represents the mean ± SD of three independent experiments.

In the same conditions (15 min reaction), GSH was not able to fully reduce the dimeric form of *At*GRXS15 (Figure 5A). Only after 1 h of incubation, GSH was able to reduce the dimeric *At*GRXS15, with 90% reduction being achieved with 10 mM GSH (Figure 5C,E). In fact, 367 ± 30 µM of GSH was needed to reduce 50% of dimeric *At*GRXS15 (Figure 5C). Concerning glutathionylated *At*GRXS15, 50% reduction was achieved with 54 ± 5 µM of GSH in 1 h (Figure 5D) and with approximately 100 µM in 15 min (Figure 5B). In both reaction times, 90% reduction was reached with approximately 1 mM GSH (Figure 5B,F).

These results are interesting in several respects. Indeed, previous studies suggested that class II GRXs lack a GSH activator site, meaning that GSH was not able to perform the reductive half-reaction, in other words, the reduction of the glutathionylated cysteine. However, the data shown here indicate that GSH concentrations below physiological levels can efficiently reduce glutathionylated *At*GRXS15. On the contrary, GSH is less efficient for the reduction of an oxidized dimeric *At*GRXS15. Part of the reason may be the lower redox potential of the intermolecular disulfide bridge. 

The second interesting observation is that both mitochondrial TRXs o from *A. thaliana* are able to reduce both oxidized forms of *At*GRXS15, with an efficiency that appears better than the one of GSH. Such a difference was already reported for yeast Grx5, but in this case, TRXs reduced an intramolecular disulfide bridge formed between the active site cysteine and the semi-conserved C-terminal cysteine, the redox potential of which is much less negative (i.e., −175 mV) [20]. In fact, even though a few TRXs were reported to catalyze deglutathionylation reactions, they are present in different compartments and from different sources, yeast, microalga, plant, plathelminths, or mice [37,38,39,40,41].

Finally, these in vitro results may have another implication. Indeed, because *At*GRXS15 is the only GRX reported in plant mitochondria but is unable to reduce roGFP2 in vitro using GSH, it is surprising that a mitochondrially-expressed roGFP2 is reduced [26,27]. Hence, from our results, we might hypothesize that *At*TRXs o contribute to the recycling of glutathionylated *At*GRXS15 in cellulo, thus allowing to maintain roGFP2 reduced.

## 4. Conclusions

In this study, we investigated the redox properties of *At*GRXS15. We observed that, despite the sole cysteine residue has an acidic pKa as in other class I or II GRXs, the oxidized states formed in vitro upon GSNO, H_2_O_2_, and GSSG treatments are different because of the absence of the semi-conserved C-terminal cysteine. Two oxidation forms with quite electronegative redox midpoint potentials, *At*GRXS15 homodimers and glutathionylated monomers, are formed and reduced by GSH and mitochondrial *At*TRXs o, albeit not with the same efficiency. Interestingly, we observed that mitochondrial TRXs o from Arabidopsis possess deglutathionylation activity as reported already for cytosolic *At*TRX h [37]. Hence, the combined presence of *At*GRXS15 and *At*TRXs o may account for the reduction of a mitochondrion-expressed roGFP2 but also possibly other glutathionylated target proteins. In conclusion, given the potential oxidative environment existing in mitochondria, *At*GRXS15 may become transiently oxidized, which may actually slow down Fe-S cluster insertion in some proteins during adverse conditions. The reduction of these reversible oxidation forms would allow the rapid recovery of the capacity to bind an Fe-S cluster and promote the maturation of Fe-S proteins, including those present in the respiratory chain complexes and in lipoate synthase [42].

## Data Availability

All data are included in the article.
